# Semantic Evidential Grid Mapping Using Monocular and Stereo Cameras †

**DOI:** 10.3390/s21103380

**Published:** 2021-05-12

**Authors:** Sven Richter, Yiqun Wang, Johannes Beck, Sascha Wirges, Christoph Stiller

**Affiliations:** 1 Institute of Measurement and Control Systems, Karlsruhe Institute of Technology (KIT), Engler-Bunte-Ring 21, 76131 Karlsruhe, Germany; eliaswangyiqun@gmail.com (Y.W.); sascha.wirges@kit.edu (S.W.); stiller@kit.edu (C.S.); 2 Atlatec GmbH, Haid-und-Neu-Straße 7, 76131 Karlsruhe, Germany; jbeck@atlatec.de

**Keywords:** autonomous driving, environment perception, grid mapping, stereo vision, monocular vision

## Abstract

Accurately estimating the current state of local traffic scenes is one of the key problems in the development of software components for automated vehicles. In addition to details on free space and drivability, static and dynamic traffic participants and information on the semantics may also be included in the desired representation. Multi-layer grid maps allow the inclusion of all of this information in a common representation. However, most existing grid mapping approaches only process range sensor measurements such as Lidar and Radar and solely model occupancy without semantic states. In order to add sensor redundancy and diversity, it is desired to add vision-based sensor setups in a common grid map representation. In this work, we present a semantic evidential grid mapping pipeline, including estimates for eight semantic classes, that is designed for straightforward fusion with range sensor data. Unlike other publications, our representation explicitly models uncertainties in the evidential model. We present results of our grid mapping pipeline based on a monocular vision setup and a stereo vision setup. Our mapping results are accurate and dense mapping due to the incorporation of a disparity- or depth-based ground surface estimation in the inverse perspective mapping. We conclude this paper by providing a detailed quantitative evaluation based on real traffic scenarios in the KITTI odometry benchmark dataset and demonstrating the advantages compared to other semantic grid mapping approaches.

## 1. Introduction

Environment perception modules in automated driving aim at solving a wide range of tasks. One of these is the robust and accurate detection and state estimation of other traffic participants in areas that are observable by on-board sensors. For risk assessment of the current scene, information about unobservable areas is also important. Furthermore, drivable areas must be perceived in order to navigate the automated vehicle safely. To reduce computational power, a common framework for solving all of these tasks is desirable. Additionally, it is preferable to use multiple heterogeneous sensors to increase the robustness of the whole system. In the literature, occupancy grid maps are frequently considered, as they enable the detection of other traffic participants while additionally modeling occlusions due to their dense grid structure. Most of the presented methods only include the processing of range sensor measurements such as Lidar and Radar and solely model occupancy without semantic states. Cameras have received less attention in the past couple of years as Lidar sensors have become more and more affordable. However, compared to cameras, Lidar sensors are still more expensive. Furthermore, cameras are superior when it comes to understanding semantic details in the environment. In [1], we presented a semantic evidential fusion approach for multi-layer grid maps by introducing a refined set of hypotheses that allows the joint modeling of occupancy and semantic states in a common representation. In this work, we use the same evidence theoretical framework and present two improved sensor models for stereo vision and monocular vision that can be incorporated in the sensor data fusion presented in [1].

**Figure 1:**
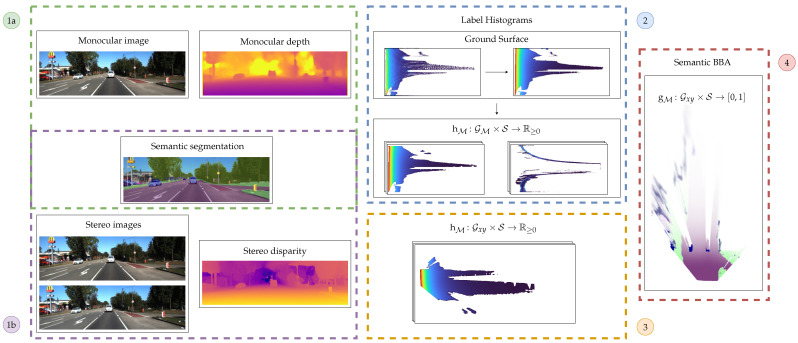
Overview of the described grid mapping framework. On the front end, both monocular images are processed to obtain depth maps (**1a**) or stereo images are used to estimate a disparity map (**1b**). Both of them are accompanied by a pixelwise semantic segmentation image. The images are used as input for a label histogram calculation in a setup-dependant grid in the second step (**2**). This label histogram is transformed into a cartesian grid (**3**) and finally transformed into a semantic evidential grid map (**4**).

In the remainder of this section, we briefly introduce the terms of the Dempster–Shafer theory (Section 1.1) relevant to this work. We then review past publications on stereo vision-based and monocular vision-based grid mapping, monocular depth estimation and semantic grid mapping in Section 1.2, followed by highlighting our focus for the proposed methods in Section 1.3. In Section 2, we give an overview of our semantic evidential models and the multi-layer grid map representations. We further describe our proposed semantic evidential grid mapping pipelines, depicted in Figure 1, in detail. We evaluate our processing steps based on challenging real traffic scenarios and compare the results of both methods in Section 3. Finally, we conclude this paper and give an outlook to future work in Section 4.

### 1.1. Dempster–Shafer Theory of Evidence (DST)

The Dempster–Shafer theory of evidence (DST), originally introduced in [2], is an extension of Bayes theory and provides a framework to model uncertainty and combine evidence from different sources. For the hypotheses set of interest Ω, called frame of discernment, the basic belief assignment (BBA)

$$m:2^{\Omega }\to \left[0,1\right]\;,\;m\left(\emptyset \right)=0,\;\sum_{A\in 2^{\Omega }}m\left(A\right)=1$$

assigns belief masses to all possible combinations of evidence. In contrast to probability measures, the BBA does not define a measure in the measure theoretical sense as it does not satisfy the additivity property. In consequence, the belief mass assigned to the whole set Ω models the amount of total ignorance explicitly. Based on a BBA, lower and upper bounds for the probability mass Pr(·) of a set $A\in 2^{\Omega }$ can be deduced as

$$\sum_{B\subseteq A}m\left(B\right)=BA\leq \mathrm{Pr}\left(A\right)\leq pl(A)=\sum_{B\cap A\neq \emptyset }m\left(B\right),$$

where bel(·) and pl(·) are called belief and plausibility, respectively.

### 1.2. Related Work

Occupancy grid maps, as introduced by Elfes et al. in [3], are often used for dense scene state estimation as they enable explicit modeling of free space and occlusion. While cartesian grid maps are especially suitable for fusing measurements over time as, e.g., presented in [4,5], other coordinate systems are more suitable for modeling sensor characteristics. In [6], Badino et al. compared different tessellations; namely, cartesian, polar and u-disparity grids for modeling free space in stereo-based occupancy grid maps. Perrollaz et al. use a u-disparity grid to estimate a stereo-based occupancy grid map and further considered different measurement models for assigning pixel measurements to grid cells in [7]. Danescu et al. used the grid representation from [8] to estimate a dynamic occupancy grid map with a low-level particle filter in [9]. Yu et al. proposed in [10] to model free space in a v-disparity grid and occupancy in a u-disparity grid before combining both in a stereo-based occupancy grid map using an evidence theoretical framework. As opposed to all previously mentioned works that considered occupancy and free space only, Giovani et al. added one occupancy refinement value denoting the semantic state to their grid map representation in [11]. However, as they did not include the semantics in their evidential hypotheses set, well-established combination rules could not be applied. Recent work on stereo-based grid mapping has been published by Valente et al. in [12] and Thomas et al. in [13]. While Valente et al. only modeled occupancy in a u-disparity grid with a subsequent fusion with Lidar-based occupancy grid maps in the cartesian space, Thomas et al. incorporated semantic hypotheses in an evidential framework. Focusing on estimating a road model, however, the hypotheses set they considered is limited to the static world.

Semantic grid mapping has also been exploited based on measurements from monocular cameras. Erkent et al. estimated in [14] semantic grid maps by fusing pixel-wise semantically labeled images with Lidar-based occupancy grid maps in a deep neural network. Lu et al. directly trained a variational encoder–decoder network on monocular RGB images to obtain a semantic top-view representation in [15]. Both networks result in a semantic grid map representation containing one class per grid cell, thus discarding knowledge about the label estimation distribution and uncertainty.

For transforming measurements from the image domain to a top-view representation, a pixelwise depth estimation is needed. In the last few years, tremendous progress in monocular depth estimation has been witnessed, especially after the wide deployment and improvement of deep neural networks. There are three main approaches for monocular depth estimation with deep neural networks: supervised depth prediction from RGB images, self-supervised (unsupervised) depth prediction with monocular videos and self-supervised depth completion.

Nowadays, with the help of convolutional neural networks (CNN), the results, such as in [16,17,18], have become superior to previous works in terms of speed and accuracy. However, the resolution of the monocular depth estimation in those papers is relatively low. To overcome this predicament, Alhashim et al. present a convolutional neural network for computing a high-resolution depth map given a single RGB image with the help of transfer learning [19]. All the above methods attempt to directly predict each pixel’s depth in an image using models that have been trained offline on a colossal training dataset with the ground truth images of depth information. While these methods have enjoyed great success, to date, they have been restricted to scenes where extensive image collections and their corresponding pixel depths are available. For the case without depth ground truth dataset, an unsupervised learning framework is presented in [20] for the task of monocular depth and camera motion estimation from unstructured video sequences. In [21], the authors generate disparity images from monocular images by training the network with an image reconstruction loss and stereo images training dataset, exploiting epipolar geometry constraints. Finally, Qiao et al. tackle the inverse projection problem in [22] by jointly performing monocular depth estimation and video panoptic segmentation. With their method, they are able to generate 3D point clouds with instance-level semantic estimates for each point.

### 1.3. Goals of This Work

This work aims to provide two accurate and efficient grid mapping frameworks. One is based on stereo cameras and the other one is based on a monocular camera. In contrast to many past publications on vision-based grid mapping like [6,7,8,10,12], we use a wide range of different semantic classes, which can be provided by vision. Instead of assigning only one semantic label per grid cell as in [11,14,15], we use the hypotheses set introduced in [1] to model uncertainty for eight semantic hypotheses in a consistent evidential framework. In order to achieve a dense and smooth BBA for ground hypotheses, we make use of encapsulated ground surface estimations to approximate the pixel-to-area correspondence in the top-view space. The resulting semantic evidential multi-layer grid map can then be fused with range sensor-based grid maps, as described in [1].

## 2. Materials and Methods

In this section, we summarize the underlying evidential models, introduce our multi-layer grid map representations in Section 2.1 and introduce all coordinate systems used throughout the mapping pipeline in Section 2.2. Then we introduce how to get the input images for the label histogram calculation in Section 2.3, followed by a detailed description of the label histogram calculation in the u-disparity and u-depth space in Section 2.4. We further explain how the label histogram is transformed to a cartesian grid in Section 2.5. We conclude this section by presenting the calculation of the BBA based on the label histogram in Section 2.6.

### 2.1. Semantic Evidential Framework

The frame of discernment

Ω={c,cy,p,om,nm,s,sw,t}

consists of the hypotheses car (*c*), cyclist (cy), pedestrian (*p*), other movable object (om), non movable object (nm), street (*s*), sidewalk (sw) and terrain (*t*). This hypotheses set can be seen as a refinement of the classical occupancy frame consisting of the two hypotheses occupied and free by considering the hypotheses sets

$$O=\left\{\left\{c\right\},\left\{cy\right\},\left\{p\right\},\left\{om\right\},\left\{nm\right\}\right\}\subset 2^{\Omega }$$

and

$$F=\left\{\left\{s\right\},\left\{sw\right\},\left\{t\right\}\right\}\subset 2^{\Omega }.$$

This makes it particularly suitable for the fusion of semantic estimates with range measurements in top-view as outlined in [1]. For the BBA, we consider the hypotheses set consisting of singletons

$$S=\left\{\left\{c\right\},\left\{cy\right\},\left\{p\right\},\left\{om\right\},\left\{nm\right\},\left\{s\right\},\left\{sw\right\},\left\{t\right\}\right\}\subset 2^{\Omega }$$

as all hypotheses combinations are either conflicting by definition or not estimated by the semantic labeling. We define the two-dimensional grid $G=P_{1}\times P_{2}$ on the rectangular region of interest $R=I_{1}\times I_{2}\subset R^{2}$, where

$$\begin{array}{cc} P_{i} & =\{I_{i,k},\;k\in \left\{0,\cdots ,s_{i}-1\right\}\},\\ I_{i,k} & =[o_{i}+k\;\delta_{i},o_{i}+\left(k+1\right)\;\delta_{i}),\;i\in \left\{1,2\right\} \end{array}$$

forms a partition of the interval $I_{i}$ with equidistant length $\delta_{i}\in R$, origin $o_{i}\in R$ and size $s_{i}\in N$. The BBA m on $2^{\Omega }$ is then represented by the multi-layer grid map

$$\begin{array}{cc} g_{M}:G\times S & \to [0,1],\\ \left(C,\omega \right) & \mapsto m_{C}\left(\omega \right), \end{array}$$

where $m_{C}$ is the corresponding BBA in the grid cell C∈G.

### 2.2. Coordinate Systems

We use four coordinate systems in our processing chain. The first is the image coordinate system with coordinates $\left(u,v\right)\in R^{2}$ rectified according to a pinhole camera model. For mapping stereo vision measurements to the top-view, the u-disparity coordinate system with coordinates $\left(u,d\right)\in R^{2}$ is used as an intermediate representation in order to be able to model disparity estimation errors explicitly. When depth is estimated directly as in most of the monocular vision-based methods, a u-depth coordinate system with coordinates $\left(u,z\right)\in R^{2}$ is used. For the final grid representation, a cartesian coordinate system with coordinates $\left(x,y\right)\in R^{2}$ is used. To indicate the corresponding coordinate system, the considered region of interest is subscripted as $R_{uv}$, $R_{ud}$, $R_{uz}$ and $R_{xy}$, respectively. The same notation is used for the attached grids $G_{uv}$, $G_{ud}$, $G_{uz}$ and $G_{xy}$. Furthermore, we introduce the mappings

$$\begin{array}{cc} T_{uv}^{ud} & :R_{uv}\to R_{ud},\;T_{ud}^{xy}:R_{ud}\to R_{xy},\\ T_{uv}^{uz} & :R_{uv}\to R_{uz},\;T_{uz}^{xy}:R_{uz}\to R_{xy} \end{array}$$

for transforming coordinates from one system to another.

### 2.3. Input Representation

We define a stereo vision measurement

$$M_{\mathrm{stereo}}=\left\{\left\{P_{i}\in \;G_{uv},\;i\in \left\{1,\cdots ,n\right\}\right\},\left\{f_{\mathrm{sem}},f_{\mathrm{disp}},f_{{\mathrm{conf}}_{\mathrm{disp}}}\right\}\right\},$$

as a tuple of a set of pixels $P_{i}$ and the three images

$$f_{\mathrm{sem}}:G_{uv}\to S,\;f_{\mathrm{disp}}:G_{uv}\to R,\;f_{{\mathrm{conf}}_{\mathrm{disp}}}:G_{uv}\to \left[0,1\right]$$

which is the pixel-wise semantic labeling image $f_{\mathrm{sem}}$, the disparity image $f_{\mathrm{disp}}$ and disparity confidence image $f_{{\mathrm{conf}}_{\mathrm{disp}}}$.

In the case of measurements stemming from a monocular camera, the disparity image $f_{\mathrm{disp}}$ is replaced by the depth image $f_{\mathrm{depth}}:G_{uv}\to R$:

$$M_{\mathrm{mono}}=\left\{\left\{P_{i}\in \;G_{uv},\;i\in \left\{1,\cdots ,n\right\}\right\},\left\{f_{\mathrm{sem}},f_{\mathrm{depth}},f_{{\mathrm{conf}}_{\mathrm{depth}}}\right\}\right\}$$

**Figure 2:**
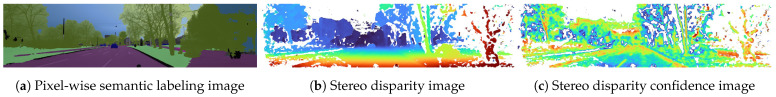
The three input images to our stereo vision-based grid mapping pipeline used in [23]. © 2021 IEEE. Reprinted, with permission, from Richter, S.; Beck, J.; Wirges, S.; Stiller, C. Semantic Evidential Grid Mapping based on Stereo Vision. In Proceedings of the 2020 IEEE International Conference on Multisensor Fusion and Integration for Intelligent Systems (MFI), Karlsruhe, Germany, 14–16 September 2020, pp. 179–184, doi: 10.1109/MFI49285.2020.9235217.

Note that the confidence images may be set to one for all pixels in case the disparity or depth estimation does not output one. In this case, every pixel is attached the same weight in the subsequent grid mapping pipeline. Figure 2 shows an example for the stereo vision measurements that were used in [23].

### 2.4. Label Histogram Calculation

As introduced in [1], we calculate the BBA based on the label histogram, which resembles the contribution of accumulated pixels to a class in a given grid cell. We use a u-disparity grid and a u-depth grid to compute the label histogram for stereo vision-based and monocular vision-based grid mapping, respectively. These discretization spaces have the advantage that disparity and depth estimation uncertainty can be modeled explicitly, as, e.g., outlined in [8]. For the sake of simplicity, we subsequently refer to the measurement grid as $G_{M}\in \left\{G_{ud},G_{uz}\right\}$. The label histogram

$$h_{M}:G_{M}\times S\to R_{\geq 0}$$

of the measurement M on the u-disparity grid or the u-depth grid is given by

$$h_{M}\left(C,\omega \right)=\sum_{P\in G_{uv}}w_{\omega }\left(C,P\right)\;{𝟙}_{\left\{\omega \right\}}\left(f_{\mathrm{sem}}\left(P\right)\right),$$

where $w_{\omega }$ is a window function specifying the contribution of the measurement based on the pixel $P\in G_{uv}$ to the cell $C\in G_{M}$ and

$${𝟙}_{X}\left(x\right)=\left\{\begin{array}{cc} 1, & \mathrm{if}\;x\in X,\\ 0, & \mathrm{else}, \end{array}\right.$$

denotes the indicator function. We apply different measurement models depending on the assigned semantic hypothesis. For the object hypotheses ω∈O, we treat each pixel measurement *P* as a point measurement p∈P that is the center coordinate of the pixel *P*. We then calculate the window function $w_{\omega }$ based on the inverse sensor model Pr(X∈C|p) to model spatial uncertainty. Here, *X* denotes the random variable modeling the actual position that the pixel measurement *P* is based on. The window function further contains the confidence $f_{{\mathrm{conf}}_{k}}$ of the corresponding range estimate k∈{disp,depth}. Assuming statistical independence between the spatial uncertainty and the uncertainty of the range estimate, the window function $w_{\omega }$ for object classes ω∈O is finally set to

$$w_{\omega }\left(C,P\right)=\mathrm{Pr}\left(X\in C\;|\;p\right)\;\cdot \;f_{{\mathrm{conf}}_{k}}\left(P\right).$$

**Figure 3:**
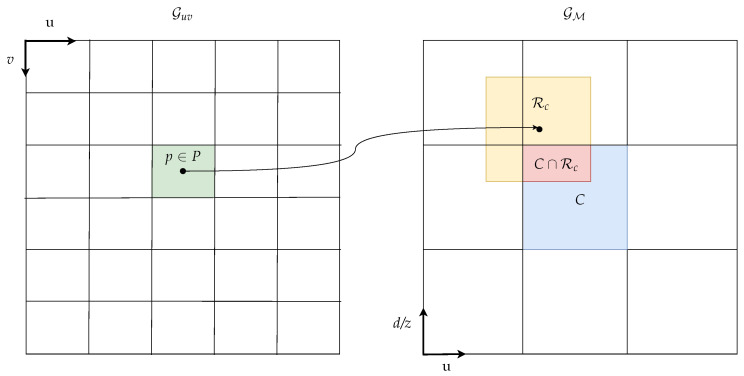
Mapping of measurements with assigned object labels from image to u-disparity/-depth grid.

In order to keep the computational complexity at a minimum, we assume *X* to be uniformly distributed in a rectangle $R_{c}$ centered around $c=T_{uv}^{M}\left(p\right)\in R_{M}$ with size *d* such that

$$\mathrm{Pr}\left(X\in C\;|\;p\right)=\frac{\mu (C\cap R_{c})}{\mu (R_{c})},$$

where μ(·) is the two-dimensional Lebesgue measure. The mapping of pixels with assigned object labels is sketched in Figure 3.

Treating pixels with assigned object labels as points is a simplification based on a lack of knowledge about object surfaces. For pixels labeled as ground, however, the surface can be assumed to be locally planar. We use this prior knowledge and propose an approximating pixel-to-area correspondence to obtain dense mapping results for the ground hypotheses. The label histogram $w_{\omega }$ for the ground hypotheses ω∈F is given by

(1) $$w_{\omega }\left(C,P\right)=\frac{1}{\mu (C)}\int_{C}\left(f_{X}*{𝟙}_{A_{P}}\right)\left(x\right)\;dx,$$

where $f_{X}$ is the probability density function of the random variable *X* modeling the measurement position and

$$A_{P}=T_{uv}^{ud}\left(P\right)\subset R_{M}$$

is the area in the grid space corresponding to the measurement pixel *P*. For pixels classified with ground labels, the shape of this area depends on the ground surface. We approximate the resulting label histogram for ground hypotheses by approximating $A_{P}$ with rectangles based on an encapsulated ground surface estimation in the three steps: ground estimation, pixel area approximation and area integral calculation.

#### 2.4.1. Ground Surface Estimation

A ground surface estimation is obtained based on the current image measurements in two stages. First, the height is averaged over all pixels that correspond to a given grid cell *C* with an assigned ground label and disparity or depth value exceeding a given threshold. Here, each pixel is treated as a point measurement leading to sparse mapping results, especially at far distances. Furthermore, the disparity or depth estimate might add further sparsity depending on the used method. The quality of the stereo disparity estimation based on pixel matching, for example, heavily relies on the local contrast of the camera image. This leads to poor disparity estimation results in smooth areas, especially on the ground, which results in no height being computed here. The sparse ground estimation is augmented in the second stage using the inpainting method introduced in [24]. This inpainting algorithm is based on the Navier–Stokes equations for fluid dynamics and matches gradients at inpainting region boundaries. To avoid large errors, the inpainting is only done in a neighborhood of the sparse ground estimation defined by the inpainting mask $f_{\mathrm{mask}}$. This mask is computed as

$$f_{\mathrm{mask}}\left(P\right)={𝟙}_{\left[0,T_{1}\right]}\left(D_{1}\left(P\right)\right)\;\cdot \;{𝟙}_{\left[0,T_{2}\right]}\left(D_{2}\left(P\right)\right),$$

where $D_{1}$ and $D_{2}$ are the distance transforms based on the masks defining the observed and unobserved image regions, respectively, and $T_{1},T_{2}\in R$ are thresholds defining the interpolation neighborhood. We justify the application of this data augmentation by the assumption that gradient jumps in the height profile would lead to gradient jumps in the pixel intensity and thus imply well-estimated disparity and depth. Consequently, the inpainted regions are restricted to areas without jumps in the height profile.

#### 2.4.2. Pixel Area Approximation

Depending on the ground surface relief, a pixel patch may correspond to arbitrarily shaped areas $A_{P}$ in the grid space. Given a grid cell $C\in G_{ud}$, the label histogram from Equation (1) for the ground label ω∈F contains the sum of all pixel portions overlapping with the cell projected into the image domain. To accelerate the mapping process, we approximate the projected cell by a rectangle $R_{P}$. Utilizing the estimated ground height, the homogeneous position in cartesian coordinates $c^{\prime }={\left(x,y,z,1\right)}^{T}$ can be computed based on the lower left cell corner point $c_{ll}$ and the upper right cell corner point $c_{ur}$ in u-disparity space. The lower left sub-pixel $\left(u_{ll},v_{ll}\right)$ and the upper right sub-pixel coordinates $\left(u_{ur},v_{ur}\right)$ are then computed using the perspective mapping F as

$$\left(u_{i},v_{i},d_{i}\right)=F\left(c_{i}^{\prime }\right)=K\;\cdot \;c_{i}^{\prime },\;i\in \left\{ll,ur\right\},$$

where *K* is the 3×4 pinhole camera matrix. Based on the projected corner points, the projected rectangle is then given by

$$R_{P}=\left[u_{ll},u_{ur}\right)\times \left[v_{ll},v_{ur}\right).$$

**Figure 4:**
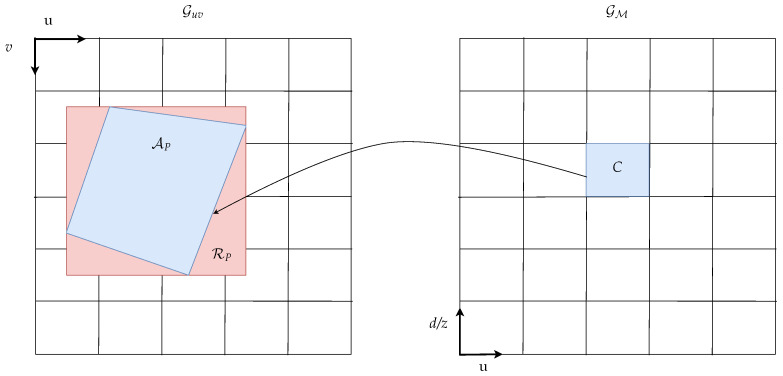
Mapping of measurements with assigned ground labels from image to u-disparity/-depth grid.

The grid cell approximation is depicted in Figure 4.

#### 2.4.3. Area Integral Calculation

Finally, the label histogram approximation for the ground labels ω∈F can efficiently be calculated based on the integral image

$${\bar{f}}_{\omega }\left(I_{1,k},I_{2,l}\right)=\sum_{i=0}^{k}\sum_{j=0}^{l}{𝟙}_{\left\{\omega \right\}}\left(f_{\mathrm{sem}}\left(I_{1,i},I_{2,j}\right)\right)$$

as

$$\begin{array}{cc} h_{M}\left(c,\omega \right) & \approx {\bar{f}}_{\omega }\left(u_{ur},v_{ur}\right)-{\bar{f}}_{\omega }\left(u_{ur},v_{ll}\right)\\ \; & -{\bar{f}}_{\omega }\left(u_{ll},v_{ur}\right)+{\bar{f}}_{\omega }\left(u_{ll},v_{ll}\right). \end{array}$$

Note that in the upper equation, $u_{i}$ and $v_{i}$ are sub-pixel coordinates and the corresponding integral image ${\bar{f}}_{\omega }$ is evaluated using bilinear interpolation.

### 2.5. Grid Transformation to Cartesian Space

**Figure 5:**
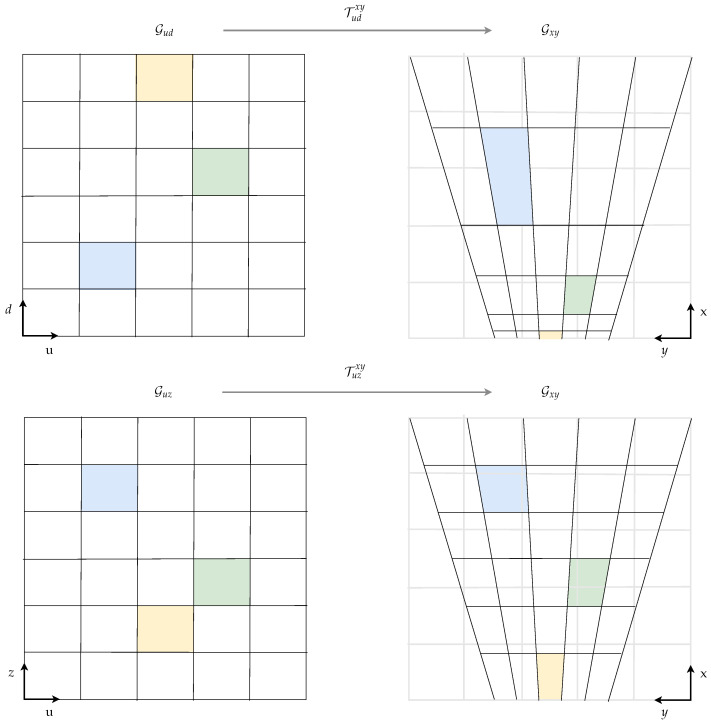
In the cartesian grid on the right-hand side, the grid cells are influenced by the distorted overlayed areas based on the corresponding u-disparity or u-depth grid cell, respectively.

The label histogram grid map layers $h_{M}\left(\;\cdot \;,\omega \right)$ are transformed from u-disparity/-depth space to the cartesian space before the BBA calculation to prevent inconsistencies in the belief assignment due to interpolation artifacts. Note that cartesian grid cells close to the camera, for instance, correspond to many u-disparity grid cells, while one u-disparity grid cell covers several cartesian grid cells at far distances. The relations between the considered tessellations are sketched in Figure 5. Non-regular cell area correspondences occur not only between u-disparity/-depth and cartesian grids. Yguel et al. investigated this effect in detail for the switch from a polar to cartesian coordinate system in [25]. Simple remapping methods lead to the so-called Moiré effect due to undersampling, which is well known in computer graphics. We tackle this issue by applying a well-established upsampling principle in relevant areas. By analyzing the area ratio between a cartesian cell and the corresponding u-disparity/-depth cell, a set of points is chosen lying on an equidistant grid within the cell. The u-disparity/-depth coordinate is calculated for each point, and the label histogram value is computed based on the u-disparity/-depth grid map utilizing bilinear interpolation. The label histogram’s final value for the cartesian cell is a weighted average over all sampled cell points.

### 2.6. Basic Belief Assignment

The label histogram is subsequently used to compute a consistent BBA in the measurement grid map $g_{M}$. The BBA is computed based on the false-positive probability $p_{\omega }$ as

$$g_{M}\left(C,\omega \right)=\prod_{\theta \in \Omega \backslash \left\{\omega \right\}}p_{\theta }^{h_{M}\left(C,\theta \right)}\left(1-p_{\omega }^{h_{M}\left(C,\omega \right)}\right),$$

for the relevant hypotheses ω∈S. Note that $p_{\omega }$ can easily be determined based on the confusion matrix of the evaluation data set of the semantic labelling network.

## 3. Results

**Figure 6:**
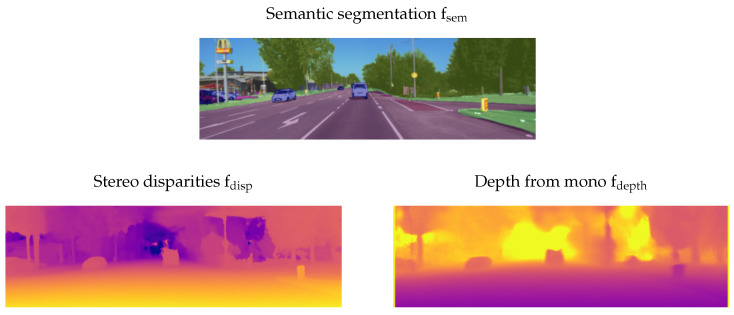
Results of the three neural networks used to generate the input images for our proposed grid mapping pipeline.

We execute our proposed method based on two setups using the Kitti odometry benchmark [26]. In the first case, we calculate stereo disparities based on the two color cameras in the Kitti sensor setup using the guided aggregation net for stereo matching presented by Zhang et al. in [27]. The authors connect a local guided aggregation layer that follows a traditional cost filtering refining thin structures to a semi-global aggregation layer. In the second setup, we only use the left color camera and compute a depth map using the unsupervised method presented by Godard et al. in [21]. Both neural networks are openly available on GitHub and have been trained or at least refined using the Kitti 2015 stereo vision benchmark. For calculating the pixelwise semantic labeling, the neural network proposed by Zhu et al. in [28] was used. Their network architecture is openly available as well and achieves a mean intersection over union (IoU) of 72.8% in the Kitti semantic segmentation benchmark. Note that all of the above choices were made independently of runtime considerations. In both cases, the pixelwise confidences for depth and disparity, respectively, are set to one as the corresponding networks do not output adequate information. In Figure 6, an example of the three used input images is depicted.

The region of interest of our cartesian grid map is 100 m in *x*-direction and 50 m in *y*-direction where the sensor origin is located at (0 m, 25 m). The cell size is 10 cm in both dimensions.

In the remainder of this section, we first present the ground truth that we used to evaluate our method in Section 3.1. We then present some visual results in Section 3.2. Finally, we present a detailed quantitative evaluation in Section 3.3.

### 3.1. Ground Truth Generation

**Figure 7:**
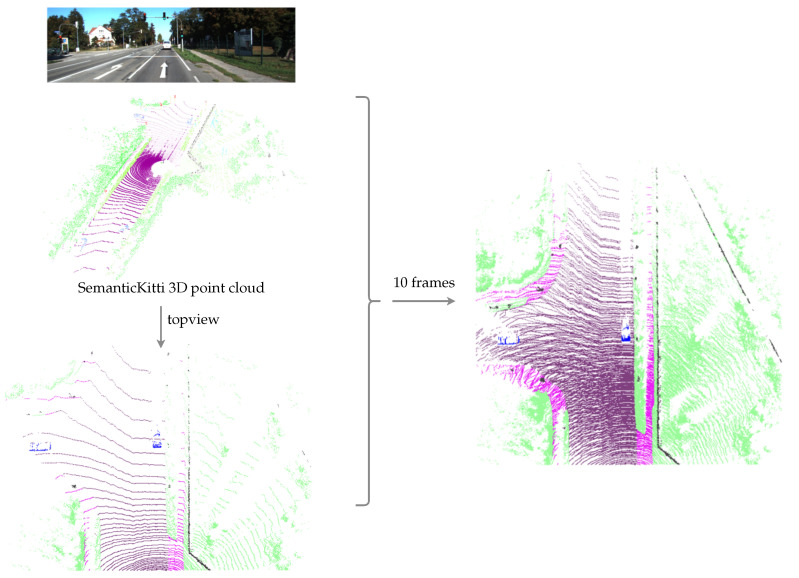
The generation of the ground truth used for the quantitative evaluation. Three-dimensional semantic point clouds from ten frames are merged and mapped into a top-view grid.

We base our quantitative evaluation on the SemanticKITTI dataset presented by Behley et al. in [29]. SemanticKitti extends the Kitti odometry benchmark by annotating the 360° Lidar scans with semantics labels using a set of 28 classes. Here, we merge those classes to obtain semantic labels corresponding to our singleton hypotheses S. Using the labeled poses in the Kitti odometry dataset, the point clouds from ten frames are transformed into the current pose, compensating for ego-motion and the subsequent accumulation. This densifies the semantic point cloud around the ego vehicle. The thus accumulated 3D semantically annotated point cloud is mapped into the same grid $G_{xy}$ that is used for our semantic evidential grid map representation. The generation of our ground truth is illustrated in Figure 7. When using multiple frames to build a denser ground truth, some cells covered by dynamic objects are covered by road pixels. In order to remove those conflicts, we use morphological operations to remove the ground labels in those regions, as can be seen in Figure 7 at the locations of the two vehicles that are present in the depicted scene. Subsequently, the grid map containing the ground truth labels is denoted as

$$g_{\mathrm{GT}}:G_{xy}\to S\cup \left\{\mathrm{unknown}\right\},$$

assigning both a semantic label or the label “unknown” to each grid cell $C\in G_{xy}$.

### 3.2. Visual Evaluation

**Figure 8:**
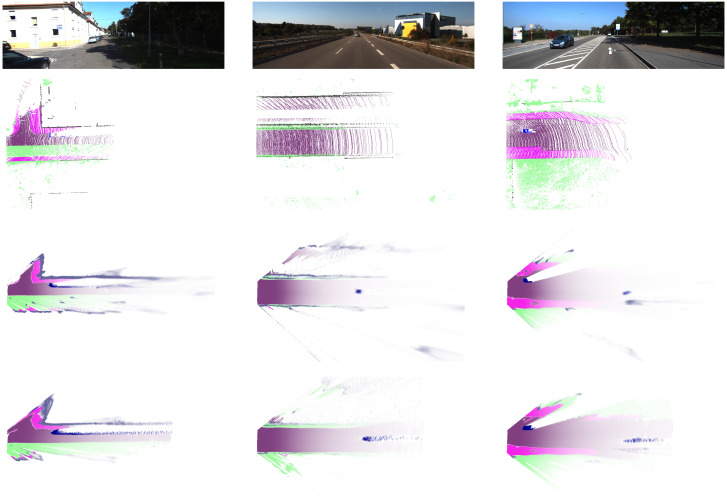
The resulting BBA for stereo and mono images. Each column corresponds to one frame in the Kitti odometry benchmark depicted in the image in the first row. The second row shows the ground truth, the third row shows the results for stereo vision, and the last row shows the results for monovision.

We process the first 1000 frames for the sequences 00 to 10 in the Kitti odometry dataset. Figure 8 depicts visual impressions of the results for the sequences 00, 01, and 02. The first thing that stands out is that the detection range in both the mono- and stereo-based grid maps surpasses the one in the Lidar-based ground truth. The BBA in our resulting evidential grid maps decreases with the distance to the sensor origin, which aligns with the intuition that the uncertainty is higher at larger distances. The first scenario in the left column was captured in a suburban region in Karlsruhe and contains a series of residences on the left, a t-crossing to the left, and a sidewalk on the right that is separated from the road by terrain. The border of the residences appears to be captured better when using the stereo pipeline. As the sidewalk on the right is covered by shadows leading to low contrast in the corresponding image region, its geometry cannot be captured with both pipelines. The middle column shows a highway scenario with a vehicle in front of the ego vehicle at about 50 m distance. The guardrails in both the ego vehicle and the opposite lane can only be captured using the stereo pipeline. The leading vehicle is detected more precisely using the stereo pipeline as well. In the third column, a scenario in a rural area with a vehicle passing on the opposite lane is depicted. There is a sidewalk on each side of the road with adjacent terrain. The rough geometry of the parts can be captured in both the mono and the stereo pipeline. The passing vehicle is detected better using the stereo pipeline, whereas its shape is slightly distorted using the monocular vision pipeline due to higher inaccuracy in the depth estimation. As a general observation, it can be stated that the errors in both camera-based reconstructions are dominated by flying pixels at object boundaries that result from inconsistencies between the pixelwise semantic estimate and the depth or disparity estimation.

### 3.3. Quantitative Evaluation

We provide a quantitative evaluation of our method based on the intersection over union per class and the overall ratio of correctly predicted cell states.

#### 3.3.1. Intersection over Union

The intersection over union (IoU) and the mean intersection over union (mIoU) are defined as

$${\mathrm{IoU}}_{\omega }=\frac{{\mathrm{TP}}_{\omega }}{{\mathrm{TP}}_{\omega }+{\mathrm{FP}}_{\omega }+{\mathrm{FN}}_{\omega }},\;\mathrm{mIoU}=\frac{1}{\left|S\right|}\sum_{\omega \in S}{\mathrm{IoU}}_{\omega },$$

where ${\mathrm{TP}}_{\omega }$ presents the number of true positive cells, ${\mathrm{FP}}_{\omega }$ the number of false-positive cells, and ${\mathrm{FN}}_{\omega }$ the number of false-negative cells of the label ω∈S. In this context, a grid cell is considered as a true positive if the class in the ground truth coincides with the class ω∈S that has been assigned the highest BBA. Note, hence, that this metric does not consider the measure of uncertainty that is encoded in the BBA. Therefore, we calculate the modified intersection-over-union metrics

$${\mathrm{IoU}}_{\omega }^{\prime }=\frac{{\mathrm{TP}}_{\omega }^{\prime }}{{\mathrm{TP}}_{\omega }^{\prime }+{\mathrm{FP}}_{\omega }^{\prime }+{\mathrm{FN}}_{\omega }^{\prime }},\;{\mathrm{mIoU}}^{\prime }=\frac{1}{\left|S\right|}\sum_{\omega \in S}{\mathrm{IoU}}_{\omega }^{\prime },$$

based on the modified rates

$${\mathrm{TP}}_{\omega }^{\prime }=\sum_{C\in G_{xy}}{𝟙}_{\left\{g_{\mathrm{GT}}\left(C\right)\right\}}\left(\omega \right)\;g_{M}\left(C,\omega \right),$$

$${\mathrm{FP}}_{\omega }^{\prime }=\sum_{C\in G_{xy}}{𝟙}_{\left\{S\backslash g_{\mathrm{GT}}\left(C\right)\right\}}\left(\omega \right)\;g_{M}\left(C,\omega \right),\;{\mathrm{FN}}_{\omega }^{\prime }=\sum_{\phi \in S\backslash \omega }{\mathrm{FP}}_{\phi }^{\prime }.$$

Table 1 and Table 2 show the above-defined IoU metrics for the sequences 00 to 10 in the Kitti odometry benchmark. The tables contain the numbers for all considered semantic classes except for other movable objects (om) as it barely occurs in the test sequences. The stereo vision pipeline outperforms the monocular vision pipeline for almost all classes. This is expected as the used stereo disparity estimation is more accurate than the monocular depth estimation. In general, the numbers for both setups are in similar regions as the ones presented in the Lidar-based semantic grid map estimation from Bieder et al. in [30]. They reach a 39.8% mean IoU with their best configuration. Our proposed method reaches 37.4% and 41.0% mean IoU in the monocular and stereo pipeline, respectively. The accuracy for small objects as pedestrians and cyclists is very low as small errors in the range estimations have a high effect compared to the objects size. Comparing the numbers for ${\mathrm{mIoU}}_{\omega }$ with ${\mathrm{mIoU}}_{\omega }^{\prime }$ incorporating the BBA, it stands out that the modified IoU is significantly higher. For the modified IoU, means of 44.7% and 48.7% are reached in the two setups. The reason for this is that higher uncertainties in wrongly classified cells lower the modified false-positive and false-negative rates ${\mathrm{FP}}_{\omega }^{\prime }$ and ${\mathrm{FN}}_{\omega }^{\prime }$ and thus also the denominator in the calculation of ${\mathrm{mIoU}}_{\omega }^{\prime }$. The results show that wrong classifications are attached with a higher uncertainty and that the BBA can be used as a meaningful measure for uncertainty.

#### 3.3.2. Ratio of Correct Labels

As a second class of metrics, we consider the ratio of correctly classified cells

$$\mathrm{CR}=\frac{\sum_{C\in G_{xy}}T_{C}}{\sum_{C\in G_{xy}}\left(T_{C}+F_{C}\right)},$$

where $T_{C}\in \left\{0,1\right\}$ equals one if the correct label ω∈S was assigned the highest BBA greater than zero and $F_{C}\in \left\{0,1\right\}$ is one if the highest BBA greater than zero corresponds to the wrong label. The counterpart incorporating the BBA reads

$${\mathrm{CR}}^{\prime }=\frac{\sum_{C\in G_{xy}}T_{C}m_{C}}{\sum_{C\in G_{xy}}\left(T_{C}+F_{C}\right)m_{C}},\;m_{C}=\underset{\omega \in S}{arg max}g_{M}\left(C,\omega \right).$$

We have calculated ${\mathrm{CR}}^{\prime }$ as well as CR for sequences 00 to 10. The results are presented in Table 3 for the stereo vision pipeline and in Table 4 for the monocular vision pipeline. The numbers confirm the tendencies collected in the IoU-based evaluation. The modified ratios ${\mathrm{CR}}^{\prime }$ based on the BBA are higher than the ones that are based solely on one predicted class per cell and the ratios of the stereo vision pipeline are slightly above the ones of the monocular vision pipeline. Besides the consistency between range estimation and semantic segmentation, the quality of the semantic segmentation itself naturally influences the final results strongly. We found that the majority of the errors in the segmentation occur in the distinction between the road and the sidewalk. Experiments showed that ${\mathrm{CR}}^{\prime }$ can be improved by up to 10% depending on the sequence when merging the two classes. Besides the Lidar-based semantic top-view maps presented in [30], we can compare our results to the hybrid approach using Lidar and RGB images from Erkent et al. presented in [14]. They achieve a ratio of correctly labeled cells of 81% in their best performing setup, indicating that our approach performs slightly better. However, note that they predict a different set of classes without uncertainty considerations.

## 4. Conclusions

We presented an accurate and efficient framework for semantic evidential grid mapping based on two camera setups: monocular vision and stereo vision. Our resulting top-view representation contains evidential measures for eight semantic hypotheses, which can be seen as a refinement of the classical occupancy hypotheses free and occupied. We explicitly model uncertainties of the sensor setup-dependant range estimation in an intermediate grid representation. The mapping results are dense and smooth, yet not complete as no estimates are given in unobserved areas. In our quantitative evaluation, we showed the benefits of our evidential model by obtaining significantly better error metrics when considering the uncertainties. This is one of the main advantages of our method compared to other publications and enables our pipeline to perform comparably well to competitive ones using more expensive sensors such as Lidar [14,30]. The second advantage is the underlying semantic evidential representation that makes fusion with other sensor types as range sensors straight forward, see [1]. The main bottlenecks in our pipeline are the semantic segmentation and the range estimation in the image domain as well as the consistency between both. Especially the influence of the latter might easily be underestimated as inconsistencies of a few pixels already imply large distortions at higher distances.

In future work, we will focus on developing a refinement method to improve the consistency between range and semantic estimation in the image domain. In this regard, it might also be promising to combine both in a mutual aid network to achieve a higher consistency in the first place. We will then fuse the presented vision-based semantic evidential grid maps with evidential grid maps from range sensors based on the method described in [1]. Furthermore, we will incorporate the fused grid maps into a dynamic grid mapping framework that is able to both accumulate a semantic evidential map as well as track dynamic traffic participants. Finally, we aim at providing a real-time capable implementation of our framework by utilizing massive parallelization on state-of-the-art GPUs.

## Figures and Tables

**Table 1:** Class IoUs ${\mathrm{IoU}}_{\omega }$ (${\mathrm{IoU}}_{\omega }^{\prime }$) for the stereo vision pipeline in %. The dash indicates that there are no corresponding objects in the sequence. The column on the right contains the mean IoUs mIoU (${\mathrm{mIoU}}^{\prime }$).

Seq.	Car	Cyclist	Pedestrian	Non Movable	Street	Sidewalk	Terrain	⌀
00	51.0 (65.7)	5.4 (6.3)	3.4 (4.7)	40.6 (50.9)	92.3 (95.6)	64.4 (72.7)	29.0 (35.3)	40.9 (47.3)
01	22.8 (45.8)	10. 3(8.8)	-	27.0 (36.6)	85.3 (92.8)	-	59.2 (66.5)	29.2 (35.8)
02	48.9 (66.7)	3.0 (2.9)	0.3 (0.3)	17.4 (23.8)	86.5 (91.4)	49.8 (60.3)	56.2 (57.0)	37.4 (43.2)
03	33.8 (54.1)	2.0 (3.2)	-	26.2 (33.6)	84.7 (88.4)	60.5 (67.0)	82.6 (85.2)	41.4 (47.4)
04	45.7 (64.0)	-	-	26.7 (31.0)	90.1 (92.9)	34.9 (43.7)	64.2 (67.4)	37.4 (42.7)
05	43.6 (60.4)	3.3 (5.2)	6.0 (8.2)	32.4 (43.3)	88.8 (93.1)	57.7 (67.5)	20.0 (23.5)	36.0 (43.0)
06	31.7 (49.1)	4.2 (4.9)	1.5 (1.6)	28.0 (39.8)	80.8 (88.9)	50.2 (62.3)	79.1 (84.4)	39.4 (47.3)
07	44.2 (61.6)	5.7 (7.1)	15.6 (17.8)	43.0 (52.3)	89.3 (93.7)	61.9 (69.4)	71.7 (76.2)	47.3 (54.0)
08	37.5 (56.1)	8.3 (12.4)	6.6 (10.2)	33.5 (46.7)	87.1 (91.9)	57.0 (67.1)	72.1 (75.2)	43.2 (51.4)
09	37.7 (57.7)	5.0 (6.1)	5.7 (15.7)	29.3 (41.7)	85.4 (90.7)	53.4 (64.9)	60.3 (65.7)	39.5 (49.0)
10	33.4 (50.8)	-	4.6 (7.2)	28.2 (36.4)	80.6 (85.2)	45.3 (52.5)	48.8 (53.5)	34.4 (40.8)
all	40.8 (59.0)	5.0 (6.7)	5.5 (8.1)	30.7 (41.4)	85.9 (91.3)	54.2 (64.2)	65.1 (69.9)	41.0 (48.7)

**Table 2:** Class IoUs ${\mathrm{IoU}}_{\omega }$ (${\mathrm{IoU}}_{\omega }^{\prime }$) for the monocular vision pipeline in %. The dash indicates that there are no corresponding objects in the sequence. The column on the right contains the mean IoUs mIoU (${\mathrm{mIoU}}^{\prime }$).

Seq.	Car	Cyclist	Pedestrian	Non Movable	Street	Sidewalk	Terrain	⌀
00	40.6 (51.5)	3.5 (3.6)	0.4 (0.7)	42.4 (59.1)	89.9 (92.8)	60.4 (71.2)	30.7 (43.3)	38.3 (46.0)
01	15.4 (25.0)	7.1 (9.0)	-	18.3 (13.1)	82.8 (90.9)	-	63.4 (72.6)	26.7 (30.1)
02	35.6 (50.4)	3.2 (3.9)	0.1 (0.1)	16.1 (22.6)	84.7 (88.9)	46.8 (54.9)	56.9 (60.8)	34.8 (40.2)
03	21.9 (31.7)	2.1 (3.4)	-	16.9 (18.9)	79.6 (83.8)	49.2 (55.9)	75.6 (81.8)	35.0 (39.4)
04	15.6 (30.6)	-	-	31.6 (36.4)	86.9 (90.3)	35.0 (41.2)	65.1 (70.6)	33.5 (38.4)
05	25.5 (39.5)	4.5 (5.6)	1.6 (2.6)	31.4 (45.6)	86.9 (90.4)	54.2 (65.1)	20.3 (34.1)	32.1 (40.4)
06	19.2 (30.4)	4.0 (5.0)	1.0 (1.7)	32.3 (49.0)	77.8 (84.8)	46.7 (57.9)	79.6 (86.0)	37.2 (45.0)
07	32.5 (46.5)	5.3 (6.4)	4.3 (7.4)	44.0 (57.9)	85.9 (90.3)	57.6 (68.2)	70.5 (81.0)	42.9 (51.1)
08	24.9 (37.8)	6.7 (8.8)	1.6 (2.0)	35.8 (54.2)	85.0 (89.1)	53.4 (62.8)	71.0 (77.9)	39.8 (47.5)
09	26.1 (40.8)	2.6 (2.7)	3.8 (7.1)	32.9 (44.9)	83.5 (88.4)	49.5 (60.6)	62.4 (72.1)	37.3 (45.2)
10	19.9 (31.7)	-	3.0 (3.9)	25.2 (34.4)	76.2 (80.6)	40.7 (48.1)	47.4 (61.2)	30.4 (37.1)
all	27.7 (41.2)	4.2 (5.2)	2.0 (3.0)	29.5 ( 41.7)	83.2 (88.3)	49.9 (59.6)	65.0 (74.1)	37.4 (44.7)

**Table 3:** Ratio of correctly labeled grid cells for the stereo vision pipeline.

Seq.	00	01	02	03	04	05	06	07	08	09	10	All
CR	81.5	81.1	78.6	86.7	83.7	73.6	82.5	83.9	82.9	79.3	73.4	80.8
${\mathrm{CR}}^{\prime }$	87.0	87.9	84.2	89.3	87.9	81.3	88.2	88.4	87.8	85.9	78.8	86.2

**Table 4:** Ratio of correctly labeled grid cells for the monocular vision pipeline.

Seq.	00	01	02	03	04	05	06	07	08	09	10	All
CR	80.3	81.0	77.7	81.5	83.1	71.9	81.9	81.6	81.5	78.9	70.1	79.3
${\mathrm{CR}}^{\prime }$	87.8	88.9	83.8	86.3	87.7	83.4	88.1	88.3	87.5	86.2	78.5	86.3
